# Quantification of the volume fraction of fat, water and bone mineral in spongiosa for red marrow dosimetry in molecular radiotherapy by using a dual-energy (SPECT/)CT

**DOI:** 10.1016/j.zemedi.2022.01.005

**Published:** 2022-03-12

**Authors:** Maikol Salas-Ramirez, Michael Lassmann, Johannes Tran-Gia

**Affiliations:** Department of Nuclear Medicine, University of Würzburg, Würzburg, Germany

**Keywords:** DECT, Quantitative computed tomography, Bone volume fraction, Fat volume fraction, Water volume fraction, Molecular radiotherapy, Red marrow dosimetry

## Abstract

A patient-specific absorbed dose calculation for red marrow dosimetry requires quantifying patient-specific volume fractions of the red marrow, yellow marrow, and trabecular bone in the spongiosa of several skeletal sites. This quantification allows selecting appropriate *S* values calculated from the parameterized radiation transport models for bone and bone marrow dosimetry. Currently, no comprehensive, individualized, and non-invasive procedure is available for quantifying the volume fractions of red marrow, yellow marrow, and trabecular bone in the spongiosa. This study aims to provide a new quantitative method based on dual-energy computed tomography to fill this gap in red marrow dosimetry using a (SPECT/)CT system.

**Methods:**

First, a method for parametrizing the photon attenuation coefficients relative to water was implemented. Next, a method to calculate the effective atomic number (*Z*_*eff*_) and effective mass density (*ρ*_*eff*_) using dual-energy CT (DECT) was employed. Lastly, two- and three-material decomposition using a dual-energy quantitative CT method (DEQCT) was performed in an anthropomorphic spine phantom and two bone samples of a boar, respectively.

The measurements of *Z*_*eff*_ and *ρ*_*eff*_ were compared with the *syngo.CT DE Rho/Z* tool (Siemens Healthineers). Furthermore, the DEQCT method implemented in this study (DEQCT-I) was compared with a second DEQCT method based on the use of external material standards (DEQCT-II). DEQCT-II was used as reference method for calculating relative errors.

**Results:**

The two-material decomposition in the anthropomorphic spine phantom presented a maximum relative error of −10% for the bone mineral density quantification. Furthermore, *Z*_*eff*_ and *ρ*_*eff*_ calculated by DEQCT-I differed from *syngo.CT DE Rho/Z tool* by less than 4.4% and 1.9%, respectively. The three-material decomposition in the two bone samples showed a maximum relative error of 21%, −17%, and 15% for the quantification of the volume fractions of fat, water, and bone mineral equivalent materials. Lastly, *Z*_*eff*_ and *ρ*_*eff*_ calculated by DEQCT-I differed from *syngo.CT DE Rho/Z tool* by less than 8.2% and 7.0%, respectively.

**Conclusion:**

This study shows that quantifying the volume fraction of fat, water, and bone mineral using a phantom-independent and post-reconstruction DEQCT method is feasible. DEQCT-I has the advantage of not requiring prior information about the X-ray spectra or the detector sensitivity function, as is the case with spectral-based DEQCT methods. Instead, DEQCT-I, similar to other DEQCT methods depends on the chemical description of reference materials and a beam hardening correction function.

DEQCT-I method provides an individualized and non-invasive procedure using a (SPECT/)CT system to apply *S* values based on the patient-specific volume fractions of yellow marrow, red marrow, and bone mineral in red marrow dosimetry.

## 1 Introduction

In molecular radiotherapy, the improvement of red marrow dosimetry procedures has received particular attention [1], [2], mainly due to the high radiosensitivity of red marrow cells [*3*]. This leads to the red marrow as one of the potential dose-limiting tissues in molecular radiotherapy [1], [2], [4]. Furthermore, the complex spatial distribution of bone and bone marrow (red marrow plus yellow marrow) [5], [6] in the spongiosa makes red marrow dosimetry one of the most challenging dosimetry procedures [1], [7].

Red marrow dosimetry methods may be categorized into (1) blood-based and (2) imaging-based methods [1]. This classification depends on the radiopharmaceutical's localization: blood, bone (cortical and trabecular bone with the bone surface and the bone volume as sub-compartments), or marrow components [1], [7], [8], [9], [10]. A comprehensive absorbed dose calculation for the red marrow should consist of three main parts: First, the quantification of the activity uptake in the relevant compartments (blood, cortical bone [surface and volume], trabecular bone [surface and volume], and red marrow) [7]. Second, the quantification of patient-specific volume fractions of the red marrow, yellow marrow, and trabecular bone [11], [12], [13], [14], [15], [16]. Third, the selection of the appropriate S values based on a parametrized radiation transport model for bone and bone marrow dosimetry that considers as input the quantified patient-specific volume fractions of the red marrow, yellow marrow, and trabecular bone [7], [17].

In the past, our group has implemented new imaging-based methods to quantify patient-specific volume fractions of the red marrow, yellow marrow, and trabecular bone in a clinical setting [15], [16]. In a first study [16], we implemented a quantification method based on magnetic resonance imaging (MRI) to measure the volume fractions of water and fat in lumbar vertebrae as a surrogate for red and yellow marrow. In a second study [15], a dual-energy quantitative computed tomography (DEQCT) method was implemented in a dual-energy (SPECT/)CT system to measure the volume fractions of bone mineral (as surrogate for trabecular bone) and water (as surrogate for red plus yellow marrow) in the spongiosa. This study goes one step further, presenting a new post-reconstruction DEQCT method to quantify the volume fractions of bone mineral (as surrogate for trabecular bone), water (as surrogate for red marrow), and fat (as surrogate for yellow marrow) based on the parametrization of photon attenuation coefficients in the range of diagnostic X-ray energies.

## 2 Methods

CT images were acquired with a SPECT/CT hybrid system (Symbia Intevo Bold, Siemens Healthineers) with adjustable CT voltage (80, 110, and 130 kVp). The protocol DE Abdomen (80 and 130 kVp) was used for imaging all phantoms used in this study. All CT reconstructions were performed with the I41s kernel (iterative reconstruction).

The Segment Editor module of 3D Slicer (version 4.8.1) [18], [19] was used to analyze the CT images of the CT electron density phantom Model 062M (computerized imaging reference systems, CIRS) and the European Spine Phantom [20]. All mathematical calculations were performed in R (version 3.5.1) [21].

The chemical formula and density of the materials used for quantification are described in the supplemental material Table 1. The elemental mass fractions of each compound or mixture were obtained using the XCOM online tool offered by the National Institute of Standards and Technology (NIST) [22]. In case of the CIRS phantom, the manufacturer provided the elemental composition, elemental mass fractions, and mass densities of the phantom materials.

### 2.1 Implementation of the dual-energy quantitative computed tomography (DEQCT) method

The DEQCT method presented in this study applies the parametrization method of the mean photon mass attenuation coefficients introduced by Martinez et al. [23]:

(1) $${\bar{\mu }}_{m,E_{1,2}}^{*}=\frac{{\bar{\mu }}_{E_{1,2}}^{*}}{\rho_{e}^{*}}=\alpha \left(E_{1,2}\right)\cdot \left(1-Z_{eff}^{*n}\right)+Z_{eff}^{*n}$$

Here, the symbol * denotes values relative to water (${\bar{\mu }}_{m,E_{1,2}}^{*}={\bar{\mu }}_{m,E_{1,2}}/{\bar{\mu }}_{m,H_{2}O,E_{1,2}}^{}$, $\rho_{e}^{*}=\rho_{e}/\rho_{e,H_{2}O}$, $Z_{eff}^{*n}=Z_{eff}^{n}/Z_{eff,H_{2}O}^{n}$), *E*_1,2_ corresponds to the effective energy of an equivalent mono-energetic beam for each poly-energetic CT X-ray beam used in this study (80 and 130 kVp), *α*(*E*_1,2_) and *n* are constants characteristic of the CT X-ray beam, and $\rho_{e}^{*}$ is the electron density relative to water of a compound or mixture:

(2) $$\rho_{e}^{*}=\frac{N_{A}\cdot \rho_{m}\cdot \sum w_{i}\cdot Z_{i}/A_{i}}{N_{A}\cdot \rho_{H_{2}O}\cdot \left(w_{H}\cdot Z_{H}/A_{H}+w_{O}\cdot Z_{O}/A_{O}\right)}=\frac{\rho_{e,compound}}{\rho_{e,H_{2}O}}$$

*N*_*A*_ is the Avogadro number, $w_{i}$ is the elemental weight of the *i*th element in the compound, and *A*_*i*_ and *Z*_*i*_ are the atomic mass and atomic number of the *i*th element in the compound, respectively. The subscripts H and O correspond to the hydrogen and oxygen atoms of the water molecule, respectively.

*Z*_*eff*_ corresponds to the effective atomic number of a compound and is defined as:

(3) $$Z_{eff}={\left(\frac{\sum_{i}w_{i}Z_{i}^{n+1}/A_{i}}{\sum_{i}w_{i}Z_{i}/A_{i}}\right)}^{1/n}$$

#### 2.1.1 Calculation of *n* and *α*(*E*_1,2_)

The value of *n* was calculated using the methodology by Martinez et al. [*23*], which is based on the calculation of the sum of the residuals resulting from the linear fit of $\left(\frac{{\bar{\mu }}_{E_{j}}^{*}}{\rho_{e}^{*}}-Z_{eff}^{*n}\right)\;\text{vs.}\;\left(1-Z_{eff}^{*n}\right)$ for energies between 50 and 100 keV in steps of 1 keV and for *n* between 2 and 4 in steps of 0.01 (200 data points). To calculate *α*(*E*_1,2_), two CT acquisitions (80 and 130 kVp) of the CIRS phantom with seven material inserts (adipose tissue, breast, muscle, liver, and three different bone qualities: 200, 800, and 1250 mg/cm^3^ of hydroxyapatite) were performed. Table 1 shows the corresponding acquisition parameters. Next, the average Hounsfield units (HU) of all materials were determined in a segmentation analysis using 3D Slicer [18], [19]. In addition, the HU values of both datasets (80 and 130 kVp) were converted into a mean linear attenuation coefficient relative to water divided by the electron density relative to water (${\bar{\mu }}_{m,E_{1,2}}^{*}$):

(4) $${\bar{\mu }}_{m,E_{1,2}}^{*}=\frac{{\bar{\mu }}_{E_{1,2}}^{*}}{\rho_{e}^{*}}=\frac{\rho_{e,H_{2}O}}{\rho_{e,phantom\;material}}\left(\frac{HU_{E_{1,2}}}{1000}+1\right)$$

**Table 1:** CT acquisition parameters.

Phantom	CT System	Voltage (kVp)	Effective mAs: (mAs)	Collimation (mm)	Pitch
CIRS	(SPECT/)CT Intevo Bold	80	100	16 × 0.6	1.2
		130	28		

ESP	(SPECT/)CT Intevo Bold	80	12	16 × 0.6	1.2
		130	12		

Boar Bones	(SPECT/)CT Intevo Bold	80	51	16 × 0.6	1.2
		130	16		

Lastly, the values of *α*(*E*_1,2_) were obtained as the slope of the linear fit of $\left(\frac{{\bar{\mu }}_{E_{1,2}}^{*}}{\rho_{e}^{*}}-Z_{eff}^{*n}\right)\;\text{vs}.\;\left(1-Z_{eff}^{*n}\right)$ for both datasets (80 and 130 kVp).

#### 2.1.2 Calculation of the effective atomic number and effective mass density of a material mixture using DEQCT

To calculate the effective atomic number (*Z*_*eff*_) and the effective mass density of a mixture (*ρ*_*eff*_), we followed the steps described by Heismann et al. [24] and Liu et al. [25]:

2.1.2.1. A look-up table was generated to calculate the atomic number of a compound or mixture:

(5) $$\frac{{\bar{\mu }}_{m,E_{1}}^{*}}{{\bar{\mu }}_{m,E_{2}}^{*}}=\frac{{\bar{\mu }}_{E_{1}}^{*}}{{\bar{\mu }}_{E_{2}}^{*}}=F\left(Z\right)$$

It was calculated using Eq. (1), and it connects *F*(*Z*) and *Z* for all elements between *Z* = 1 and *Z* = 30 for both X-ray kilovoltages.

2.1.2.2. A column associated to the effective electron density was added to the look-up table:

(6) $$\rho_{e,eff}^{*}\left(Z\right)=\frac{{\bar{\mu }}_{eff,E_{1}}^{*}}{{\bar{\mu }}_{m,E_{1}}^{*}}$$

While ${\bar{\mu }}_{m,E_{1}}^{*}$ is obtained from Eq. (1), ${\bar{\mu }}_{eff,E_{1}}^{*}$ is obtained directly from the CT image, considering:

(7) $${\bar{\mu }}_{eff,E_{1,2}}^{*}=\frac{{\bar{\mu }}_{eff,E_{1,2}}}{{\bar{\mu }}_{H_{2}O,E_{1,2}}}=\frac{HU_{E_{1,2}}}{1000}+1$$

Here, HU is the average Hounsfield Unit in volumes of interest (VOI), which were drawn for each mixture.

2.1.2.3. Calculation of *Z*_*eff*_ and *ρ*_*eff*_:

*Z*_*eff*_ and *ρ*_*eff*_ were calculated based on the look-up table from steps 1 and 2. By using Eq. (7) for both CT images, the values of ${\bar{\mu }}_{eff,E_{1}}^{*}$ and ${\bar{\mu }}_{eff,E_{2}}^{*}$ were obtained. Next, Eq. (5) provided the values *F*(*Z*_*eff*_) as the ratio ${\bar{\mu }}_{eff,E_{1}}^{*}/{\bar{\mu }}_{eff,E_{2}}^{*}$. Lastly, *Z*_*eff*_ was obtained by the interpolation of the closest values of *Z* and *F*(*Z*) in the look-up table. To calculate *ρ*_*eff*_, a linear fit between *ρ*_*eff*_ and $\rho_{e,eff}^{*}\left(Z_{eff}\right)$ for a set of materials with known mass density is necessary. Therefore, the values *Z*_*eff*_ and $\rho_{e,eff}^{*}\left(Z_{eff}\right)$ for the seven phantom materials of the CIRS phantom were calculated using the look-up table (steps 1 and 2). Subsequently, $\rho_{eff}\left(\rho_{e,eff}^{*}\right)$ was obtained by a linear fit between the mass densities and the measured electron density of all seven material samples.

#### 2.1.3 Calculation of the volume fraction using DEQCT

Liu et al. [25] proposed a mass fraction conservation method. In continuation, our goal is to quantify the volume fractions of bone-, water- and fat-equivalent tissues in the spongiosa. For reasons of volume fraction conservation, we assumed hydroxyapatite (bone mineral), fat, and water in the mixture to be immiscible [15]. Consequently, we converted mass fraction into volume fraction by using the expression [25]:

(8) $$VF_{X}=\frac{\rho_{eff}\cdot MF_{X}}{\rho_{X}}$$

Here, *ρ*_*X*_ is the mass density of the compound *X* (e.g., hydroxyapatite, oil, water), *ρ*_*eff*_ is the effective density of the mixture, and *VF*_*X*_, and *MF*_*X*_ are the volume fraction and mass fraction of compound *X*, respectively. From this conversion, the three-equation system proposed by Liu et al. [25] can be reformulated in terms of the volume fractions of the materials in the mixture:

(9) $$\left\{\begin{array}{l} {\bar{\mu }}_{eff,E_{1}}^{*}=C\left(Z_{eff}\right)\cdot \left(VF_{1}\cdot {\bar{\mu }}_{1,E_{1}}^{*}+VF_{2}\cdot {\bar{\mu }}_{2,E_{1}}^{*}+VF_{3}\cdot {\bar{\mu }}_{3,E_{1}}^{*}\right)\\ {\bar{\mu }}_{eff,E_{2}}^{*}=C\left(Z_{eff}\right)\cdot \left(VF_{1}\cdot {\bar{\mu }}_{1,E_{2}}^{*}+VF_{2}\cdot {\bar{\mu }}_{2,E_{2}}^{*}+VF_{3}\cdot {\bar{\mu }}_{3,E_{2}}^{*}\right)\\ 1=VF_{1}+VF_{2}+VF_{3} \end{array}\right)$$

Here, *VF*_1_, *VF*_2_, and *VF*_3_ are the volume fractions of the quantified materials (e.g., hydroxyapatite, fat or adipose tissue, and water or soft tissue). ${\bar{\mu }}_{X,E_{1,2}}^{*}$ is the mean linear attenuation coefficient of a specific compound (e.g., hydroxyapatite, water, oil). ${\bar{\mu }}_{1,E_{1,2}}^{*}$, ${\bar{\mu }}_{2,E_{1,2}}^{*}$ and, ${\bar{\mu }}_{3,E_{1,2}}^{*}$ were obtained by multiplying the mass attenuation coefficient relative to water (calculated by Eq. (1)) with the electron density relative to water (Eq. (2)). *C*(*Z*) is an empirical correction factor documented by Liu et al. [25] to correct for beam hardening in regions with *Z* > 10. The derivation of *C*(*Z*) is described in detail in the supplemental material.

### 2.2 Quantification of the *Z*_*eff*_ and $\rho_{e,eff}^{*}$ using DEQCT

The quantification of *Z*_*eff*_ and $\rho_{e,eff}^{*}$ by using the presented DEQCT method was tested against the nominal *Z*_*eff*_ and $\rho_{e}^{*}$ values of the CIRS material samples ($\rho_{e}^{*}/Z$ nominal values), as well as against the values obtained with the *syngo.CT DE Rho/Z* tool $\left({\left(\rho_{e}^{*}/Z\right)}_{syngo.via}\right)$ (syngo.via, Siemens Healthineers). To distinguish between the method presented in this paper and the method previously presented by Goodsitt et al. [*26*], the nomenclature DEQCT-I and DEQCT-II will be used for our new method and Goodsitt's approach [*26*], respectively.

First, the nominal *Z*_*eff*_ of the CIRS phantom materials were calculated using Eq. (3) and the value of *n* obtained in Section 2.1.1. The nominal $\rho_{e}^{*}$ were obtained using Eq. (2). The values *Z*_*eff*_ and $\rho_{e,eff}^{*}$ by using DEQCT-I were then obtained using the look-up table calculated in Section 2.1.2. Next, *Z*_*eff*_ measured using ${\left(\rho_{e}^{*}/Z\right)}_{syngo.via}$ were extracted from the generated Z-image (image exported from syngo.via [27]) using the segmented CIRS material samples from Section 2.1.2. The same procedure was applied to the $\rho_{e}^{*}\text{-image}$ (exported from syngo.via). Moreover, an intermediate step was required to obtain $\rho_{e}^{*}$ from the Rho image:

(10) $$\rho_{e}^{*}=\left(\frac{HU_{\rho_{e}^{*}-image,E_{1,2}}}{1000}+1\right)$$

Here, $HU_{\rho_{e}^{*}-image,E_{1,2}}$ is the HU measured in the $\rho_{e}^{*}\text{-image}$.

The three datasets were paired as follows: (1) DEQCT-I vs. nominal $\rho_{e}^{*}/Z$ values, (2) DEQCT-I vs. ${\left(\rho_{e}^{*}/Z\right)}_{syngo.via}$, and (3) nominal $\rho_{e}^{*}/Z$ vs. ${\left(\rho_{e}^{*}/Z\right)}_{syngo.via}$. Each of these data pairs was tested for normality and statistical significance.

### 2.3 Quantification of the bone mineral density and water in the European Spine Phantom: two material decomposition

To evaluate if our DEQCT-I method reliably performs a decomposition of two materials, measurements with the European Spine Phantom (ESP) were performed [20]. The ESP simulates three vertebrae, each consisting of three sections (volumes): (i) spongiosa, (ii) wall and endplate, and (iii) arch and processes [20]. The spongiosa volume of each vertebra (1–3) has a different mineral bone (hydroxyapatite) concentration: (1) 50 mg/cm^3^ hydroxyapatite (HA), (2) 100 mg/cm^3^ HA, and 200 mg/cm^3^ HA. Figure 3 shows the segmentation of spongiosa regions of ESP.

First, two CT images (80 and 130 kVp) of the ESP were acquired (acquisition parameters: see Table 1). Next, the three spongiosa volumes of the ESP were segmented in both CT images, and an average HU value was measured for each volume. Subsequently, for water, fat tissue [28] and hydroxyapatite, the mean linear mass attenuation coefficient (${\bar{\mu }}_{E_{1,2}}^{*}$) was calculated using the parametrization equation (Eq. (1)) in combination with the electron density relative to water (Eq. (2)) of each material.

Next, Eq. (9) was solved by setting *VF*_2_ = 0 in the third equation. Lastly, the relative errors between the bone mineral density (volume fraction multiplied by 3000 mg/cm^3^ hydroxyapatite density) quantified by the DEQCT-I method (BMD_DEQCT-I_) and the nominal bone mineral densities of the ESP (BMD_ESP_) were calculated.

Furthermore, *Z*_*eff*_ and *ρ*_*eff*_ were measured in each analyzed VOI using ${\left(\rho_{e}^{*}/Z\right)}_{syngo.via}$ and DEQCT-I. In case of ${\left(\rho_{e}^{*}/Z\right)}_{syngo.via}$, the mass density was obtained by a linear fit of the electronic density and the mass density of the analyzed phantom materials of the CT electron density phantom. The resulting linear equation was used to transform the electronic density of the analyzed VOIs in this and the next section.

### 2.4 Quantification of the volume fractions of bone mineral, fat, and water in two femurs of a boar: three material decomposition

**Figure 1:**
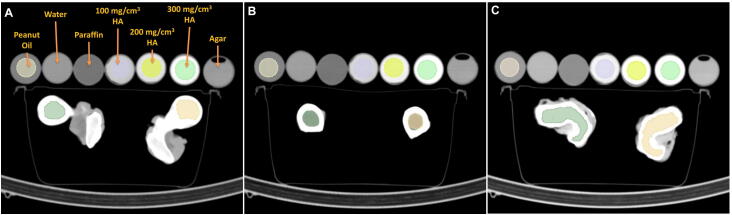
Experimental layout and bone segmentation to quantify the volume fraction bone mineral, peanut oil (fat surrogate), and mixture water/pHEMA (water surrogate).

The neck, head, and medial region of two femurs of a boar were analyzed to validate our DEQCT-I method. The two bones were placed inside a plastic container without any surrounding attenuation material. Two CT images (80 and 130 kVp) were acquired for each region (acquisition parameters: see Table 1). The image analysis was performed using the same image segmentation and quantification tools as in the previous sections. In addition to DEQCT-I, the DEQCT-II method was applied as a reference. For validation purposes, multiple calibration vials were located next to the plastic container during the measurement. The materials inside the seven calibration vials were: 100% water, 100% agar, 100% peanut oil, 100% paraffin, and three vials containing a mixture of 50% water and 50% poly(2-hydroxyethyl methacrylate) (pHEMA) mixed with different concentrations of hydroxyapatite (100, 200, and 300 mg/cm^3^) [15]. Figure 1 illustrates the positioning of the vials, the bone samples, and the corresponding volumes of interest (VOIs).

Next, each bone's neck, head, and medial region was segmented in the axial view. Similarly, the peanut oil vial and the three hydroxyapatite vials were segmented. Subsequently, for each segmented volume, an average HU value was calculated. Next, the mean linear attenuation coefficient relative to water divided by the electron density relative to water (${\bar{\mu }}_{E_{1,2}}^{*}$) was calculated for a mixture of water/pHEMA, peanut oil, and hydroxyapatite as it is described in Section 2.3. Lastly, Eq. (9) was applied to calculate the volume fractions of bone mineral, peanut oil (fat surrogate), and the mixture water/pHEMA (water surrogate). To simplify the trabecular bone composition to mineral bone only without considering the volume fraction of the organic matrix where the mineral bone is distributed could be a potential source of errors in the quantification of fat and water equivalent tissues. This simplification, however, is required to compare the DEQCT-I method with a reference method. Therefore, the materials used in DEQCT-I were selected in accordance with the external sample used by the method described by Goodsitt et al. [*26*] (DEQCT-II). Furthermore, *Z*_*eff*_ and *ρ*_*eff*_ were measured in each VOI analyzed by using ${\left(\rho_{e}^{*}/Z\right)}_{syngo.via}$ and DEQCT-I.

The HU of peanut oil, the intercept of the calibration line of the hydroxyapatite vials, and the expected HU for hydroxyapatite (obtained from the hydroxyapatite calibration line for a concentration equal to the hydroxyapatite density of 3000 mg/cm^3^) were used as equation parameters for the equation system of the DEQCT-II method. Lastly, the relative errors were calculated between the quantified volume fractions of the new DEQCT-I method (VF_BM_DEQCT-I_) and Goodsitt's DEQCT-II method (VF_BM_DEQCT-II_).

## 3 Results

### 3.1 Implementation of the Dual-energy quantitative computed tomography (DEQCT) method

**Figure 2:**
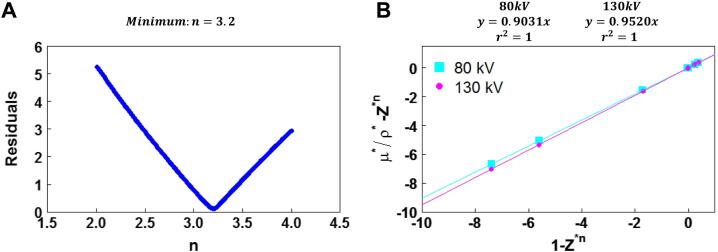
Implementation of Martinez et al. [23] parametrization method of the photon linear attenuation coefficient: (A) calculation of *n*. (B) Calculation of *α*(*E*_1,2_).

Figure 2A shows the sum of the residual resulting from the linear fit of $\left(\frac{{\bar{\mu }}_{E_{j}}^{*}}{\rho_{e}^{*}}-Z_{eff}^{*n}\right)\;\text{vs}.\;\left(1-Z_{eff}^{*n}\right)$. The minimum residual was found for *n* = 3.2, for which *α*(*E*_1_) (80 kVp CT X-ray beam) and α(*E*_2_) (130 kVp CT X-ray beam) took values of $0.903\pm 0.001\;\left(k=1\right)$ and $0.952\pm 0.001\;\left(k=1\right)$, respectively (Figure 2B). Table 2 shows the calculated and measured ${\bar{\mu }}_{m,E_{1,2}}^{*}$ values. The relative errors between calculated and measured ${\bar{\mu }}_{m,E_{1,2}}^{*}$ stayed below ± 2% for all material samples of the CIRS phantom.

**Figure 3:**
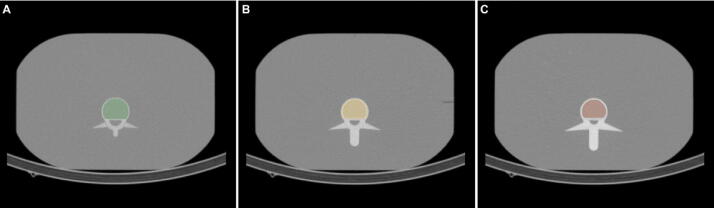
Spongiosa segmentation of the ESP: (A) vertebra 1 (50 mg/cm^3^ HA). (B) Vertebra 2 (100 mg/cm^3^ HA). (C) Vertebra 3 (200 mg/cm^3^ HA).

**Table 2:** Calculated and measured ${\bar{\mu }}_{m,E_{1,2}}^{*}$ values for the CIRS phantom materials.

Material	Measured		Calculated		% Error	
	80 kVp	130 kVp	80 kVp	130 kVp	80 kVp	130 kVp
Adipose	0.964	0.982	0.963	0.982	−0.1	0.0
Breast	0.979	0.989	0.976	0.988	−0.3	−0.1
Muscle	1.009	1.005	1.001	1.001	−0.8	−0.4
Liver	1.009	1.003	1.002	1.001	−0.7	−0.2
Bone (200 mg/cm^3^ HA)	1.189	1.095	1.167	1.083	−1.9	−1.1
Bone (800 mg/cm^3^ HA)	1.547	1.271	1.546	1.271	−0.1	0.0
Bone (1250 mg/cm^3^ HA)	1.708	1.350	1.720	1.356	0.7	0.4

Table 2 in the supplemental material presents the values of *F*(*Z*) for all chemical elements with 1 < *Z* < 30 as well as the values of ${\bar{\mu }}_{m,E_{1}}^{*}$ used to calculate the electronic densities (Eq. (5)).

### 3.2 Quantification of the effective atomic number (*Z*_*eff*_) and electronic density relative to water $\left(\rho_{e}^{*}\right)$ by using DEQCT

The statistical analysis (Shapiro–Wilk normality test) of all three data pairs (1: DEQCT-I vs. nominal $\rho_{e}^{*}/Z$ values, 2: DEQCT-I vs. ${\left(\rho_{e}/Z\right)}_{syngo.via}$, 3: nominal $\rho_{e}^{*}/Z$ vs. ${\left(\rho_{e}/Z\right)}_{syngo.via}$) showed normal distributions (*p* > 0.05). For both parameters (*Z*_*eff*_ and $\rho_{e}^{*}$), paired t-tests showed no significant differences for all data pairs with *p* > 0.51 and 0.54 (*Z*_*eff*_ and $\rho_{e}^{*}$ of data pair 1), *p* > 0.23 and 0.12 (data pair 2), and *p* > 0.23 and 0.13 (data pair 3). The values of *Z*_*eff*_ and $\rho_{e}^{*}$ are presented in the supplemental Table 3.

### 3.3 Quantification of the bone mineral density and water in spongiosa regions of the ESP: two-material decomposition

Table 3 presents *Z*_*eff*_ and *ρ*_*eff*_ of the ESP measured using ${\left(\rho_{e}^{*}/Z\right)}_{syngo.via}$ and DEQCT-I. Maximum differences of 4.4% and 1.9% were found for *Z*_*eff*_ and *ρ*_*eff*_, respectively*.*

**Table 3:** Measured (${\left(\rho_{e}^{*}/Z\right)}_{syngo.via}$) and calculated (DEQCT-I) *Z*_*eff*_ and *ρ*_*eff*_ of the spongiosa region of the European spine phantom (ESP).

Spongiosa region	syngo.CT DE Rho/Z tool		DEQCT-I		Relative error (%)	
	*Z*_*eff*_	*ρ*_*eff*_ (g/cm^3^)	*Z*_*eff*_	*ρ*_*eff*_ (g/cm^3^)	*Z*_*eff*_	*ρ*_*eff*_
Vertebra 1	8.5	1.05	8.7	1.05	2.7	0
Vertebra 2	9.2	1.08	9.5	1.1	3.6	1.9
Vertebra 3	10.1	1.16	10.5	1.18	4.4	1.7

The BMD quantification showed a maximum relative error of −10% for the spongiosa region with a bone mineral density equal to 50 mg/cm^3^; followed by 1% and −1% for the 100 and 200 mg/cm^3^ regions, respectively. Table 4 of the supplemental material shows the measured BMD and their respective relative errors.

### 3.4 Quantification of the volume fraction of bone mineral, fat, and water in two femurs of a boar: three-material decomposition

The measured (${\left(\rho_{e}^{*}/Z\right)}_{syngo.via}$) and calculated (DEQCT-I) *Z*_*eff*_ and *ρ*_*eff*_ of the spongiosa region of the ESP differed by less than 8.2% and 7.0%, respectively*.* Table 4 presents the results. The highest *Z*_*eff*_ and *ρ*_*eff*_ were found in the femoral head regions. The values of the neck region lied between the values of the head (highest values) and medial regions (lowest values).

**Table 4:** Measured (${\left(\rho_{e}^{*}/Z\right)}_{syngo.via}$) and calculated (DEQCT-I) *Z*_*eff*_ and *ρ*_*eff*_ of the spongiosa region of the boar bones.

Region	syngoCT DE Rho/Z tool		DECT-I		Relative Error (%)	
	*Z*_*eff*_	*ρ*_*eff*_ (g/cm^3^)	*Z*_*eff*_	*ρ*_*eff*_ (g/cm^3^)	*Z*_*eff*_	*ρ*_*eff*_
Head: Bone 1	11.8	1.58	12.8	1.62	7.8	2.5
Head: Bone 2	11.8	1.64	12.8	1.66	8.2	1.2
Neck: Bone 1	11.3	1.28	11.8	1.36	4.5	6.3
Neck: Bone 2	11.4	1.28	11.8	1.37	4.2	7.0
Medial: Bone 1	8.0	0.94	8.3	0.95	3.0	1.1
Medial: Bone 2	8.1	0.95	8.2	0.96	1.9	1.1

The three-material decomposition for the head region was not possible. In this region, both methods (DEQCT-I and DEQCT-II) resulted in non-physical solutions for equation system (Eq. (9)) with negative values for the peanut oil volume fraction. In these cases, the equation system was solved with the peanut oil volume fraction set to zero (*VF*_2_ = 0 in the third equation). Consequently, the quantification in the femoral head of both bones was limited to a two-material decomposition. A similar approach was followed in the medial region of the femoral bone, where both methods (DEQCT-I and DEQCT-II) resolved the equation system with negative values for the mixture of 50% water and 50% pHEMA. As before, equation 9 was solved with these volume fractions set to zero (*VF*_3_ = 0 in the third equation). The three-material decomposition was possible in the neck region of the femoral bones. In this region, Eq. (9) was solved with positive values for all three volume fractions.

Lastly, the relative errors between both DEQCT methods (DEQCT-II as reference) are presented in Table 5. The relative error for bone mineral quantification stayed below 15% in the head and neck regions and in the order of 67% in the medial region, which also presented the lowest bone mineral volume fractions (DEQCT-I: 0.01 and DEQCT-II: 0.03). The relative error associated with the fat (peanut oil) quantification stayed below 21%. Lastly, the relative error for the water (water/pHEMA) quantification stayed below −17%. Moreover, DEQCT-I overestimates the bone mineral volume (VF_BM_) (relative errors: 15% in the head, 8% in the neck) in the range of high and medium VF_BM_ (head: 0.20, neck: 0.13) and underestimates the VF_BM_ (−67% relative error) in the range of very low VF_BM_ (medial region: 0.01).

**Table 5:** Bone mineral, fat, and water volume fraction quantification in three spongiosa regions of two femoral bones of a bore.

Region	Material	DEQCT-I (volume fraction)	DEQCT-II (volume fraction)	Relative error (%)
Head Bone 1	Bone mineral	0.22	0.20	10
	Water/pHEMA	0.78	0.80	−3

Neck Bone 1	Bone mineral	0.14	0.13	8
	Peanut oil	0.41	0.34	21
	Water/pHEMA	0.45	0.54	−17

Medial Bone 1	Bone mineral	0.01	0.03	−67
	Peanut oil	0.99	0.97	2

Head Bone 2	Bone mineral	0.23	0.20	15
	Water/pHEMA	0.77	0.80	−4

Neck Bone 2	Bone mineral	0.14	0.13	8
	Peanut oil	0.38	0.33	15
	Water/pHEMA	0.48	0.54	−11

Medial Bone 2	Bone mineral	0.01	0.03	−67
	Peanut oil	0.99	0.97	2

## 4 Discussion

In this study, we propose a new DEQCT method (DEQCT-I), which allows to quantify the volume fractions of fat, water, and mineral bone in the spongiosa. DEQCT-I provides the advantage that it does not require prior knowledge about the CT detector sensitivity function and the CT X-ray source spectra (information known only by the manufacturer of the CT system), making the method feasible to implement in a clinical environment.

In the first part of this study, we calculated the values of *n* and *α*(*E*_1,2_) following the methodology by Martinez et al. [23]. In our analysis, we found an optimum *n* of 3.20, which is in agreement with the 3.21 reported in [23]. This agreement serves as proof for a correct implementation of the parametrization method. Furthermore, the values of *α*(*E*_1,2_), which were 0.903 for 80 kVp and 0.952 for 130 kVp, are close to the range of 0.919–0.948, which had been reported by Martinez et al. [23] for kilovoltages between 100 and 140 kVp for two different CT systems.

The second section corresponding to the quantification of *Z*_*eff*_ and $\rho_{e}^{*}$ by using DEQCT showed a non-significant difference between the three *Z*_*eff*_ and $\rho_{e}^{*}$ measurement methods (DEQCT-I, $\rho_{e}^{*}/Z$ nominal values, and ${\left(\rho_{e}^{*}/Z\right)}_{syngo.via}$) for the CIRS phantom materials. These results demonstrate the ability of DEQCT-I to reproduce *Z*_*eff*_ and $\rho_{e}^{*}$ values of the CIRS phantom's materials using the look-up table calculated in Section 2.1.2 of the methods.

The quantification of *Z*_*eff*_ and *ρ*_*eff*_ in the ESP showed a good agreement between DEQCT-I and ${\left(\rho_{e}^{*}/Z\right)}_{syngo.via}$. The largest relative error was observed in vertebra 3, which has the highest bone mineral concentration in the wall and the vertebral process. Therefore, the spongiosa in vertebra 3 is more susceptible to the beam hardening effect. This may explain the increase of the relative errors from vertebra 1 to vertebra 3.

The quantification of the bone mineral densities showed a good agreement and a lower relative error than a previous study [15], where we quantified bone mineral densities in the three spongiosa regions of the ESP using the same (SPECT/)CT system and a spectral-based DEQCT method, which required the CT detector sensitivity function and the CT X-ray source spectra to calculate the photon attenuation coefficients. In this previous study, we obtained relative errors of 32% (vertebra 1), 17% (vertebra 2), and 5% (vertebra 3), which are higher than the relative errors obtained in this study for DEQCT-I: −10%, −1% and 1% for vertebrae 1, 2, and 3, respectively. This decrease in relative error may be related with a better calculation of the photon attenuation coefficients using Martinez's method [23]. Furthermore, our spectral-based DEQCT method [15] had the disadvantage that we had insufficient information about the detector response function. This limitation may have introduced errors in the calculation of the photon attenuation coefficients.

The good agreement between the nominal values of the BMD in the ESP spongiosa regions and the values provided by DEQCT-I indicates that DEQCT-I performs the two-material decomposition, specifically the bone mineral quantification, with good accuracy. On the other hand, the ESP offers the advantage that the materials (bone mineral and epoxy matrix) are homogeneously distributed. This characteristic enables a better quantification of *Z*_*eff*_, *ρ*_*eff*_, and the bone mineral volume fraction. In real spongiosa tissue, where bone mineral, yellow marrow, and red marrow are heterogeneously distributed, an increase in the relative errors of *Z*_*eff*_ and *ρ*_*eff*_, as well as in the quantified volume fractions, can be expected.

Compared to the ESP, the quantification of *Z*_*eff*_ and *ρ*_*eff*_ in the boar bones showed an increase in the relative errors proportionally to *Z*_*eff*_ (see Table 4), which may be related to the beam hardening effect due to the thickness and density of the cortical bone. For *ρ*_*eff*_, we observed an increase in the relative error of the femoral neck region, where a more heterogeneous distribution of bone mineral, yellow marrow, and red marrow is expected, than in the head and medial regions. While the accuracy and exact methodology behind ${\left(\rho_{e}^{*}/Z\right)}_{syngo.via}$ are not specified by the vendor, we associate the differences between ${\left(\rho_{e}^{*}/Z\right)}_{syngo.via}$ and DEQCT-I with the beam hardening produced by the cortical bone.

The measurement in the three regions of the bore bones provided three different distribution of fat, water and bone mineral. Compared to DEQCT-II, the DEQCT-I method overestimates the bone mineral volume fraction in the range of high (head region) and medium (neck region) bone mineral volume fractions and underestimates the bone mineral volume fraction in the range of very low volume fractions (medial region). Regions such as the medial region are not expected to be measured in the human body or at least not in any of the bone sites of interest for internal dosimetry such as the axial skeleton. Therefore, based on the quantification of bone mineral in the ESP and in the head and neck bones of the boar, DEQCT-I provides a reliable measure of bone mineral.

We observed in the neck region, that DEQCT-I, in comparison to DEQCT-II, overestimates the fat volume fraction, while it underestimates the water volume fraction. The maximum relative errors for fat (peanut oil) and water (water/pHEMA) were quantified in the neck region of bone 1 (fat: 21%, water: −17%) with a volume fraction of 0.34 (fat) and 0.54 (water). In a previous, MRI-based study [16], we quantified fat and water with a relative error below 10%. Therefore, further steps are needed for improving the fat and water quantification using DEQCT-I. Moreover, some conditions in the experiment could also play a major role in the fat and water quantification. Specifically, the incapacity of DEQCT-I to perform three material decomposition in the head and medial regions of the boar bone could have the following reasons:

(1) Incomplete description of all materials located in the analyzed region: in our study, we used bone mineral (hydroxyapatite), peanut oil, and a mixture of water/pHEMA; while in the boar bone, a more complex material composition of trabecular bone (bone mineral plus organic matrix), yellow marrow, and red marrow is expected. The selection of materials used for the quantitative analysis (bone mineral, peanut oil and a mixture of water/pHEMA) was based on the necessity to apply a second DEQCT method (DEQCT-II, Goodsitt et al. [26]) for comparison. This limitation is inherent to all DEQCT methods, which require the use of reference materials (e.g. the International Commission on Radiation Units and Measurements (ICRU) tissue elemental composition [28]) to calculate the attenuation coefficients (DEQCT-I) or as external material samples [26].
(2) Simplification of the trabecular bone as mineral bone (hydroxyapatite): under this assumption, the organic matrix in which the mineral bone is distributed and the water is located in the porous bone might be considered as water- or fat-equivalent materials by both DEQCT methods (DEQCT-I and DEQCT-II). Although this approach could introduce errors in the fat and water quantification, its results can be validated without histological or μCT image analysis in contrast to the potentially more accurate approach based on the ICRU bone description [28].
(3) Fat and water material similarities: under the aspect of photon interactions, water/pHEMA (attenuation coefficients of 1.041 for 80 kVp and 1.049 for 130 kVp) is more similar to peanut oil (attenuation coefficients relative to water of 0.844 for 80 kVp and 0.861 for 130 kVp) than to bone mineral (attenuation coefficients relative to water of 6.092 for 80 kVp and 4.533 for 130 kVp). This similarity between peanut oil and water/pHEMA makes it complex to differentiate both materials especially in the presence of bone mineral.
(4) Low volume fractions of fat-equivalent tissue and water-equivalent tissue in the femoral head and the medial regions of the analyzed bones, respectively: based on *ρ*_*eff*_ measured by DEQCT-I (mean mass density in the femoral head and medial regions of 1.64 and 0.96 g/cm^3^, respectively) and the reference mass density of human cortical bone and fat tissue (1.92 and 0.95 g/cm^3^, respectively [28]), it is expected that the head and medial regions are mostly composed of bone tissue and fat, respectively. This is in agreement with the quantified volume fractions (Table 5).

### 4.1 Study limitations

The phantom geometry was limited to the vertebral geometry (ESP) and femoral bone of a boar without scattering medium. Further studies comparing DEQCT-I to 2-point Dixon MRI in bone samples could help to evaluate the accuracy of DEQCT-I in the quantification of the fat and water volume fractions. Furthermore, the use of tissue descriptions based on the ICRU [28] in the DEQCT-I method together with MRI techniques will provide information about the accuracy of DEQCT-I to measure the red marrow and yellow marrow volume fractions. Further steps to evaluate the feasibility of a three-material decomposition using DEQCT-I based on low-dose CT (typically used for attenuation correction in nuclear medicine imaging) might enable an implementation of the method as a routine step in dosimetry procedures (i.e., without the need of additional measurements and radiation exposure to the patient). Lastly, DEQCT-I has been only implemented and tested in a (SPECT/)CT system. Further steps using more sophisticated CT systems (e.g., CT systems with a dual-source, beam spectral separation by tin filters, a dual-layer detector, or photon-counting detectors [29]) could provide improvements in volume fraction quantification.

## 5 Conclusion

This study shows the feasibility of quantifying the volume fractions of fat, water, and bone mineral using a phantom-independent post-reconstruction DEQCT method. In contrast to spectral-based DEQCT methods, the presented DEQCT-I method does without any prior information about X-ray spectra or detector sensitivity function. Similar to other DEQCT methods, DEQCT-I depends on the chemical description of reference materials and a beam hardening correction function.

In animal bone sample measurements, we observed that DEQCT-I overestimates the fat volume fraction, while it underestimates the water volume fraction. Furthermore, DEQCT-I overestimates the bone mineral volume fraction in the range of high and medium bone mineral volume fractions and underestimates the bone mineral volume fraction in the range of very low volume fractions.

In conclusion, DEQCT-I provides an individualized and non-invasive procedure to determine patient-specific volume fractions of yellow marrow, red marrow, and bone mineral to be used for selection of S values in red marrow dosimetry.

## Authors’ contribution

Maikol Salas-Ramirez (MSR), Johannes Tran-Gia (JTG), and Michael Lassmann (ML) designed the study. MSR and JTG performed the experiments. MSR developed the methodology and performed the calculations. All authors analyzed and interpreted the data and wrote the manuscript.

## Funding

The study received no external funding.

## Conflict of Interest

M. Lassmann received research grants by IPSEN Pharma and Nordic Nanovector. All other authors declare no competing interests.

## Footnotes

Appendix A Supplementary data associated with this article can be found, in the online version, at https://doi.org/10.1016/j.zemedi.2022.01.005.
