# Supplementary material for: Quantification of the volume fraction of fat, water and bone mineral in spongiosa for red marrow dosimetry in molecular radiotherapy by using a dual-energy (SPECT/)CT
Source: Z Med Phys. 2022 Mar 12;32(4):428–37. doi: 10.1016/j.zemedi.2022.01.005 (PMC9948840; doi:10.1016/j.zemedi.2022.01.005)
Supplement: Supplementary file 1 [file mmc1.docx]

SUPPLEMENTAL TABLES

**Supplemental table 1**. Chemical formula and density (unit: g/cm^3^) of the material used for quantification.

| Chemical compound | Chemical formula | Density |
| --- | --- | --- |
| Hidroxyapatite | Ca_10_(PO_4_)_6_(OH)_2_ | 3.000 |
| Water | H_2_O | 1.000 |
| Mixture of water and pHEMA (Water/pHEMA) | H_2_O (50% w/v) + C_6_H_10_O_3_ (50% w/v) | 1.075 |
| Peanut oil | C_30_H_45_N_9_O_5_ | 0.910 |
| Fat tissue | ICRU report 44 ([*1*](#_ENREF_1)) | |

**Supplemental table 2**. Look-up table to quantify the effective atomic number and effective density of a mixture.

| Z | $\boldsymbol{F}\left( \boldsymbol{Z} \right)$ | ${\bar{\boldsymbol{\mu}}}_{\boldsymbol{m,}\boldsymbol{E}_{\boldsymbol{1}}}^{\boldsymbol{*}}$ |
| --- | --- | --- |
| 1 | 0.949 | 0.903 |
| 2 | 0.949 | 0.905 |
| 3 | 0.952 | 0.908 |
| 4 | 0.956 | 0.916 |
| 5 | 0.963 | 0.930 |
| 6 | 0.975 | 0.951 |
| 7 | 0.991 | 0.982 |
| 8 | 1.012 | 1.024 |
| 9 | 1.039 | 1.080 |
| 10 | 1.071 | 1.151 |
| 11 | 1.108 | 1.239 |
| 12 | 1.149 | 1.347 |
| 13 | 1.195 | 1.476 |
| 14 | 1.242 | 1.630 |
| 15 | 1.292 | 1.809 |
| 16 | 1.341 | 2.017 |
| 17 | 1.391 | 2.256 |
| 18 | 1.439 | 2.527 |
| 19 | 1.485 | 2.834 |
| 20 | 1.529 | 3.178 |
| 21 | 1.570 | 3.562 |
| 22 | 1.608 | 3.989 |
| 23 | 1.644 | 4.461 |
| 24 | 1.676 | 4.980 |
| 25 | 1.706 | 5.549 |
| 26 | 1.733 | 6.170 |
| 27 | 1.758 | 6.847 |
| 28 | 1.780 | 7.580 |
| 29 | 1.800 | 8.374 |
| 30 | 1.818 | 9.230 |

**Supplemental table 3**. Atomic number $(Z)$ and electronic densities relative to water $(\rho_{e}^{*})$ for the CIRS phantom materials measured by three different methods.

| Material | Method | $\boldsymbol{Z}_{\boldsymbol{eff}}$ | $\boldsymbol{\rho}_{\boldsymbol{e}}^{\boldsymbol{*}}$ |
| --- | --- | --- | --- |
| Adipose | DEQCT-I | 6.4 | 0.94 |
| Breast |  | 6.9 | 0.97 |
| Muscle |  | 7.6 | 1.04 |
| Liver |  | 7.7 | 1.06 |
| Bone (200 mg/cm^3^ HA) |  | 10.4 | 1.1 |
| Bone (800 mg/cm^3^ HA) |  | 13.5 | 1.44 |
| Bone (1250 mg/cm^3^ HA) |  | 14.5 | 1.69 |
| Adipose | $({\rho_{e}^{*}/Z)}_{syngo.via}$ | 6.7 | 0.94 |
| Breast |  | 7.1 | 0.96 |
| Muscle |  | 7.6 | 1.04 |
| Liver |  | 7.7 | 1.05 |
| Bone (200 mg/cm^3^ HA) |  | 9.8 | 1.1 |
| Bone (800 mg/cm^3^ HA) |  | 12.8 | 1.4 |
| Bone (1250 mg/cm^3^ HA) |  | 13.7 | 1.62 |
| Adipose | $\mathrm{nominal}\rho_{e}^{*}/Z$ values^†^ | 6.38 | 0.944 |
| Breast |  | 6.94 | 0.966 |
| Muscle |  | 7.61 | 1.036 |
| Liver |  | 7.70 | 1.055 |
| Bone (200 mg/cm^3^ HA) |  | 10.39 | 1.103 |
| Bone (800 mg/cm^3^ HA) |  | 13.47 | 1.440 |
| Bone (1250 mg/cm^3^ HA) |  | 14.46 | 1.690 |

^†^ $Z$ and $\rho_{e}^{*}$ reported with one more significant figure because the CIRS phantom manufacturer provided the elemental weights, and mass densities of the phantom materials with four significant figures.

**Supplemental table 4**. Bone mineral density quantification in the European spine phantom: two material decomposition

| Region | ESP  (BMD, mg/cm^3^) | DEQCT-I  (BMD, mg/cm^3^) | Relative Error (%) |
| --- | --- | --- | --- |
| Vertebra 1 | 50 | 45 | -10 |
| Vertebra 2 | 100 | 99 | -1 |
| Vertebra 3 | 200 | 201 | 1 |

BMD: bone mineral density

APPENDIX: DETERMINATION OF THE CORRECTION FACTOR$C(Z)$

To calculate material volume fractions in this study, it was necessary to apply an empirical correction factor $C(Z)$ for resolving the equation system (equation 8). Liu *et al*. ([*2*](#_ENREF_2)) documented the use an empirical correction factor $C(Z)$ to calculate the effective density for materials with a *Z_eff_*≥10. In our case. this correction was applied in the whole range of *Z_eff_* values measured in this study. To find the $C\left( Z \right)$ function four phantom material samples (liver. and three different bone qualities: 200 mg/cm^3^ of hydroxyapatite (HA). 800 mg/cm^3^ HA and 1.250 mg/cm^3^ HA) from the phantom CIRS model 062 were used.

1. In first step. the equation 9 was solved for the four phantom material samples considering as quantification material the phantom material samples itself (elemental composition provided by the manufacturer). fat tissue (ICRU elemental composition) ([*1*](#_ENREF_1)). and water.
2. Next. the equation 9 was solved considering ${VF}_{2}=0$ (fat tissue) in the third equation (two material decomposition: phantom material sample and water). The value of $C(Z)$ was adjusted manually to obtain a solution with a volume fraction of the phantom material sample (${VF}_{1}=1$) and a volume fraction of water equal to zero (${VF}_{3}=0$).
3. Lastly. the values of effective atomic number (measured by our DEQCT method) and the $C(Z_{eff})$ values were tabulated and adjusted to a quadratic function:

| Material | Measured $\boldsymbol{Z}_{\boldsymbol{eff}}$ | $\boldsymbol{C(}\boldsymbol{Z}_{\boldsymbol{eff}}\boldsymbol{)}$ |
| --- | --- | --- |
|  |  |  |
| Liver | 7.70 | 1.27 |
| Bone 200 mg/cm^3^ HA | 10.39 | 1.09 |
| Bone 800 mg/cm^3^ HA | 13.47 | 1.00 |
| Bone 1250 mg/cm^3^ HA | 14.46 | 0.99 |

**Fit function:**

$$C\left( Z_{eff} \right)= 6.154\times{10}^{-3}\cdot Z_{eff}^{2} - 1.775\times{10}^{-1}\cdot Z_{eff} + 2.270$$

**1.** White DR, Booz J, Griffith RV, Spokas JJ, Wilson IJ. Report 44: Tissue Substitutes in Radiation Dosimetry and Measurement. *Journal of the International Commission on Radiation Units and Measurements.* 1989;os-23.

**2.** Liu X, Yu L, Primak AN, McCollough CH. Quantitative imaging of element composition and mass fraction using dual-energy CT: three-material decomposition. *Med Phys.* 2009;36:1602-1609.
